# Discrepant molecular and clinical diagnoses in Beckwith-Wiedemann and Silver-Russell syndromes

**DOI:** 10.1017/S001667231900003X

**Published:** 2019-03-04

**Authors:** Deborah J.G. Mackay, Jet Bliek, Maria Paola Lombardi, Silvia Russo, Luciano Calzari, Sara Guzzetti, Claudia Izzi, Angelo Selicorni, Daniela Melis, Karen Temple, Eamonn Maher, Frédéric Brioude, Irène Netchine, Thomas Eggermann

**Affiliations:** 1 Faculty of Medicine, University of Southampton, Southampton SO17 1BJ, UK and Wessex Regional Genetics Laboratory, Salisbury SP2 8BJ, UK; 2 Department of Clinical Genetics, Academic Medical Center, University of Amsterdam, Amsterdam, The Netherlands; 3 Medical Cytogenetics and Molecular Genetics Laboratory, Centro di Ricerche e Tecnologie Biomediche IRCCS, Istituto Auxologico Italiano, Milan, Italy; 4 Prenatal Diagnosis Unit, Department of Obstetrics and Gynecology, ASST Spedali Civili of Brescia, Brescia, Italy; 5 Pediatric Unit, ASST Lariana Como, Como, Italy; 6 Department of Pediatrics, University “Federico II”, Napoli, Italy; 7 Department of Medical Genetics, University of Cambridge and NIHR Cambridge Biomedical Research Centre and Cancer Research UK Cambridge Centre, Cambridge Biomedical Campus, Cambridge, UK; 8 Sorbonne Université, INSERM, UMR 938, Centre de Recherche Saint-Antoine (CRSA), APHP Hôpital Trousseau, 75012 Paris, France; 9 Institute of Human Genetics, University Hospital, Technical University of Aachen, Aachen, Germany

**Keywords:** Beckwith-Wiedemann syndrome, molecular testing, Silver-Russell syndrome, unexpected results

## Abstract

Beckwith-Wiedemann syndrome (BWS) and Silver-Russell syndrome (SRS) are two imprinting disorders associated with opposite molecular alterations in the 11p15.5 imprinting centres. Their clinical diagnosis is confirmed by molecular testing in 50–70% of patients. The authors from different reference centres for BWS and SRS have identified single patients with unexpected and even contradictory molecular findings in respect to the clinical diagnosis. These patients clinically do not fit the characteristic phenotypes of SRS or BWS, but illustrate their clinical heterogeneity. Thus, comprehensive molecular testing is essential for accurate diagnosis and appropriate management, to avoid premature clinical diagnosis and anxiety for the families.

## 1. Introduction

**Fig. 1.**
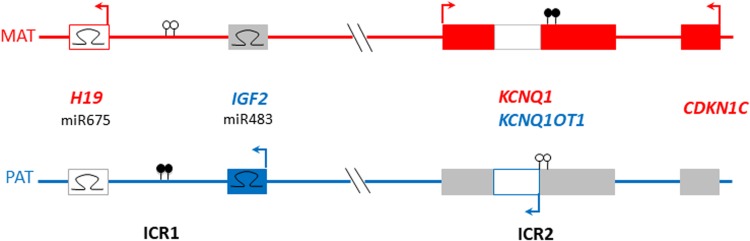
Schematic of 11p15 region indicating common imprinting disturbances (DNA methylation imbalances) associated with Beckwith-Wiedemann syndrome (BWS) and Silver-Russell syndrome (SRS). Filled lollipops: methylated imprinting control region (IC); empty lollipops: unmethylated IC; hairpins: microRNA; filled oblongs: coding genes; outline oblongs: noncoding RNA; red denotes genes expressed from maternal allele; blue denotes genes expressed from paternal allele; grey denotes genes not expressed from the allele shown.

Beckwith-Wiedemann syndrome (BWS) and Silver-Russell syndrome (SRS) are congenital imprinting disorders, associated with oppositely altered parent of origin-specific expression of two neighbouring clusters of imprinted genes on Chr11p15.5 (Soellner *et al.*, 2017 b) (Figure 1). SRS affects approximately 1:50,000 individuals, with characteristic features including pre- and post-natal growth restriction, relative macrocephaly and prominent forehead, early feeding difficulties, and body asymmetry (Wakeling *et al.*, 2016). BWS, or the recently-described Beckwith-Wiedemann Spectrum (BWSp) affects approximately 1:10,500 individuals, and its clinical features include macroglossia, anterior abdominal wall defects, prenatal and/or postnatal overgrowth, tumour predisposition and lateralized overgrowth (Brioude *et al.*, 2018). Due to their clinical heterogeneity, for both syndromes clinical scoring systems are a prerequisite for a more directed diagnostic protocol and clinical management (Wakeling *et al.*, 2016; Brioude *et al.*, 2018).

Over 50% of SRS cases are caused by loss of paternal allele methylation (LOM) of imprinting centre 1 (IC1 or H19/IGF2:IG-DMR), whereas gain of maternal allelic methylation at IC1 (GOM) can be identified in 5–10% of BWS cases. However, in BWS 50% of cases show loss of maternal allele methylation of imprinting centre 2 (IC2 or KCNQ1OT1:TSS-DMR). Sequence variants in *CDKN1C* and *IGF2*, as well as copy number variants or mosaic segmental uniparental disomy affecting chromosome 11p15.5, are also associated with BWS and SRS. In 10% of SRS patients, maternal uniparental disomy of chromosome 7 can be detected. Mosaic methylation disturbances of IC1 and IC2 are frequent (Wakeling *et al.*, 2016; Brioude *et al.*, 2018) with strong differences in distribution between different tissues (Azzi *et al.*, 2015), thereby challenging genetic testing and probably leaving several patients without molecular diagnosis. A significant fraction of children with IC1 and/or IC2 LOM have multi-locus imprinting disturbances (MLID), that is, aberrant imprinting marks at additional imprinted loci (for review see Sanchez-Delgado *et al.*, 2016).

To identify the major molecular changes, first-line testing for BWS and SRS is recommended to include DNA methylation analysis of IC1 and IC2 (Eggermann *et al.*, 2016). In fact, the majority of patients exhibit the disease-specific (epi)mutations in 11p15, but single individuals referred with symptoms consistent with BWS or SRS show molecular changes inconsistent with the clinical diagnosis, or even consistent with its molecular ‘mirror’, thus posing challenges for interpretation, diagnostic reporting and genetic counselling.

Here we describe selected cases from different European laboratories where apparent ambiguities have arisen in BWS/SRS diagnosis, to offer a precedent for interpretation and reporting. By considering the clinical data and the reason for referral and the molecular findings, we suggest to categorize these cases into three groups. Examples for each category are presented in Table 1.

**Table 1.** Cases with reported discrepancy between clinical referral and molecular diagnosis of Beckwith-Wiedemann syndrome (BWS) and Silver-Russell syndrome (SRS).

Case	Clinical referral	Asymmetry	First molecular result^1^	MLID^1^	Clinical features^2^	Reference
**A: Isolated asymmetry**						
1	BWS	Yes	IC1 LOM	No	Hemihypertrophy and facial asymmetry. Tongue left side larger than right. At WG 41: birth length 52 cm (P46), weight 3000 g (P0).	
2	BWS	Yes	IC1 LOM	No	Asymmetry of limbs. Slightly elevated AFP. At WG 40: birth weight 2570 g (P0), 47 cm (P1), OFC 33.5 cm (P5). At 1 year: height 75 cm (P15), weight 8.8 kg (P28)	
3	BWS	Yes	IC1 LOM	No	Isolated hemihypertrophy, normal growth (but shorter than her monozygous twin), methylation indices near normal in blood but lower in fibroblasts	
4	BWS	Yes	IC1 LOM	No	IUGR, relative macrocephaly, hemihyperplasia (legs, arms, kidneys) elevated AFP initially	
5	BWS	Yes	IC1 LOM	NK	Isolated hemihypertrophy	
6	BWS	Yes	IC1 LOM	NK	Features of BWS (unspecified)	
7	BWS	Yes	IC1 LOM	NK	Hemihypertrophy (clinical diagnosis reassessed as hemiatrophy after molecular diagnosis)	
8	BWS	Yes	IC1 LOM	NK	Asymmetry	
9	Asymmetry	Yes	IC1 LOM	NK	Hemihypertrophy of limbs (left leg), optic hypoplasia left eye	
10	Asymmetry	Yes	IC1 LOM	NK	Hemihypertrophy of right side	
11	Asymmetry	Yes	IC1 LOM	NK	Hemihypertrophy of limbs	
12	Asymmetry	Yes	IC1 LOM	NK	Isolated asymmetry	Russo *et al.* (2016)
13	Asymmetry	Yes	IC1 LOM	NK	Not given	
14	Asymmetry	Yes	IC1 LOM	NK	Not given	
15	Asymmetry	Yes	IC1 LOM	NK	Hemihypotrophy left arm	
16	Asymmetry	Yes	IC1 LOM	NK	Hemihypertrophy of limbs; at WG 39 birth length 48 cm, birth weight 2724 g, OFC 33 cm, ear anomalies NH-CSS: 1/6	
**B: features of SRS with or without asymmetry**						
17	SRS	Yes	IC2 LOM	NK	IUGR, micrognathia, psychomotor delay, blue sclerae	
18	SRS	Yes	IC2 LOM	No	IUGR, PNGR, relative macrocephaly, prominent forehead, triangular facies, thyroid carcinoma	
19	SRS	NK	IC2 LOM	No	Features of SRS (unspecified)	
20	SRS	NK	IC2 LOM	No	IUGR, PNGR, no relative macrocephaly, anterior midline defect, transient hypoglycaemia	Murphy *et al*. (2012)
21	SRS	No	IC2 LOM	No	IUGR, PNGR, no relative macrocephaly, developmental delay, radioulnar synostosis	Turner *et al.* (2010)
22	SRS	Yes	IC2 LOM	No	IUGR, asymmetry, feeding difficulties, excessive sweating	
23	SRS	Yes	Upd(11)pat	No	Features of SRS (unspecified)	
24	SRS	No	IC2 LOM	No	IUGR, short stature, 5th finger clinodactyly	
**C: Multi-locus imprinting disorder**						
25	BWS	No	IC1 LOM	Yes	ICSI, postnatal macrosomia, macroglossia, umbilical hernia, leg length discrepancy	Tee *et al.* (2013)
26	SRS	Yes	IC1 + IC2 LOM	Yes	IUGR (birth weight <4th centile), cleft lip and palate, feeding difficulties, mild developmental delay, behavioural difficulty	Docherty *et al.* (2015)
27	SRS	Yes	IC1 + IC2 LOM	Yes	Discordant monozygous twin. Mild IUGR and PNGR, mild motor delay, renal dysplasia	Begemann *et al.* (2011)
28	SRS	Yes	IC1 + IC2 LOM	Yes	Birth weight 2.3 kg at 40 WG: short stature, asymmetry, normal development	
29	SRS	NK	IC1 + IC2 LOM	Yes	IUGR, PNGR	
30	BWS	Yes	IC1 + IC2 LOM	Yes	Macrosomia, macroglossia, naevus flammeus, developmental delay	Begemann *et al.* (2018)
31	SRS	No	IC1 + IC2 LOM	Yes	*In vitro* fertilization, one of fraternal triplets. NH-CSS 6/6	Begemann *et al.* (2018)
32	SRS	No	IC1 + IC2 LOM	Yes	BW at 27 WG 465 g, OFC 32 cm. PNGR, respiratory support for two months, gastric tube feeding for first year. Microcephaly, precocious puberty, dysmorphism. Developmental delay. 47,XXY	Begemann *et al.* (2018)
33	BWS	No	IC2 LOM	Yes	Fetus ascertained at 16 WG with 9 mm hernia, 22 × 14 mm omphalocoele containing intestine, vacuolated placenta	
34	BWS?	No	IC2 LOM	Yes	Fetal ascertainment: induced abortion at 19 WG. Omphalocele, shortened humeri, mesenchymal placenta	Soellner *et al.* (2017 a)

1 In the majority of cases, methylation specific multiplex ligation-dependent probe amplification (MS-MLPA) based kits were used for diagnostic purposes.

2 Clinical details given at the referral of DNA samples for molecular testing. Not given: no additional clinical information provided at referral. AFP: alpha-foetoprotein; BW: birth weight; ICSI: intra-cytoplasmic sperm injection; IUGR: intrauterine growth restriction; LOM: loss of methylation; MLID: multi-locus imprinting disturbance; NH-CSS Netchine-Harbison clinical scoring system; NK: not known (clinical data not reported or molecular analysis not performed); OFC: occipitofrontal circumference; PNGR: postnatal growth restriction; upd(11)pat: paternal uniparental disomy of chromosome 11; WG: weeks of gestation.

## 2. Clinical referral of BWS or isolated asymmetry; molecular diagnosis of IC1 LOM

In three cases (patients 1, 2, 6), the initial clinical suspicion of BWS based on some key features according to the recent consensus guidelines (Brioude *et al.*, 2018) had to be revised after molecular diagnosis of a IC1 LOM. As this finding is the characteristic epimutation for SRS, two of the patients (patients 1, 2) were clinically re-evaluated, but did not fulfil the clinical Netchine-Harbison Score (NHS) for SRS (one out of six items each; Wakeling *et al.*, 2016). Interestingly, two of the patients showed more or less normal growth. In the majority of patients with IC1 LOM, asymmetry was the major symptom provoking molecular testing (e.g., cases 10–16).

Asymmetry is one of the key features of both BWS and SRS, but it can be difficult to clinically distinguish hemihypertrophy from hemihypotroply/hemiatrophy, particularly if other clinical features are lacking. Isolated lateralized overgrowth (ILO) in the presence of an 11p15 molecular anomaly is within the BWSp. ILO is sufficient to prompt BWS testing (Brioude *et al.*, 2018), and some European diagnostic laboratories have historically logged all cases of ILO for BWS first-line testing by 11p15 DNA methylation analysis. According to current consensus guidelines, isolated asymmetry is insufficient to warrant SRS testing (Wakeling *et al.*, 2016). Thus, in cases referred solely for asymmetry, identification of a molecular defect normally associated with a clinical diagnosis of SRS may be unexpected, but it is not discrepant.

## 3. Clinical features of SRS (with or without asymmetry); molecular diagnosis consistent with BWS

Some individuals with growth restriction, with or without additional SRS features, were referred for SRS diagnosis but received molecular diagnosis consistent with BWS – in the majority, IC2 LOM. Molecular SRS testing is commonly requested as an exclusion diagnosis for growth-restricted children, and in these cases, parallel testing of IC1 and IC2 occasionally diagnoses IC2 LOM. Our data confirm that IC2 LOM in BWS is not strongly associated with overgrowth (Brioude *et al.*, 2018), but that in some cases it is associated with growth restriction (Unpublished data from authors IN, FB, DJM, IKT), which when associated with body asymmetry can prompt initial clinical diagnosis of SRS. Growth restriction associated with IC2 LOM may expand the clinical spectrum of BWSp.

## 4. Clinical referral for diagnosis of SRS or BWS; molecular diagnosis of MLID

Of eight postnatal referrals with MLID, six had clinical diagnoses of SRS and two of BWS, which may reflect: (a) ascertainment bias for referrals meeting specific clinical criteria; (b) the relative likelihood of imprinting disturbance restricting rather than enhancing growth; (c) mosaic LOM in different tissues, with the critical imprinting disturbance eluding detection in the tissue analysed (Azzi *et al.*, 2015). Two cases were ascertained prenatally. One case (patient 33) was referred for 11p15.5 methylation testing after detection of omphalocele and vacuolated placenta, with normal growth parameters. Methylation specific multiplex ligation-dependent probe amplification (MS-MLPA) analysis revealed LOM of IC1, IC2 and the GNAS/GNAS-AS locus. Another case (patient 34) was ascertained with omphalocele, shortened humeri and mesenchymal placenta, and showed LOM of IC2, *GRB10* and *MEST* loci (Soellner *et al.*, 2017a). To our knowledge these are the first reported prenatal diagnoses of MLID.

MLID is detectable in approximately 25% of BWS and 10% of SRS cases with IC2 or IC1 LOM, respectively, and being mosaic by nature, may elude detection in diagnostic samples. Because MLID may result from underlying genetic changes, and may alter genetic counselling and perinatal as well as clinical management (Soellner *et al.*, 2017 a, b), it should be considered in individuals with discrepant molecular and clinical diagnoses.

## 5. Conclusion

The compilation of data from patients with unexpected molecular findings confirms the urgent need to apply comprehensive molecular tests targeting different imprinted loci to identify unexpected and/or overlapping molecular changes, and thereby to contribute to the discovery of the causative (epi)mutations in patients with unspecific phenotypes. As these examples show, the application of clinical scoring systems and the clinical evaluation can be a major prerequisite for a more directed diagnostic testing strategy, but some patients might be missed if the decision about molecular testing is strictly based on fulfilment of clinical criteria. We want to emphasize that in patients with inconclusive clinical features the communication of a clinical diagnosis should be delayed until molecular confirmation is available because a premature diagnosis might cause anxiety to the families.

The discrepancy between clinical and molecular features of BWS and SRS is an infrequent occurrence. Though objective numbers are lacking, these cases probably represent ⩽1% of diagnostic referrals. Prompt, sensitive and comprehensive molecular testing is essential for accurate diagnosis, appropriate management and genetic counselling, for these as for all imprinting disorders.

## References

[ref1] Azzi S, Steunou V, Tost J, Rossignol S, Thibaud N, Das Neves C, Le Jule M, Habib WA, Blaise A, Koudou Y, Busato F, Le Bouc Y and Netchine I. (2015). Exhaustive methylation analysis revealed uneven profiles of methylation at IGF2/IC1/H19 11p15 loci in Russell Silver syndrome. Journal of Medical Genetics 52, 53–60. 25395389 10.1136/jmedgenet-2014-102732

[ref2] Begemann M, Rezwan FI, Beygo J, Docherty LE, Kolarova J, Schroeder C, Buiting K, Chokkalingam K, Degenhardt F, Wakeling EL, Kleinle S, González Fassrainer D, Oehl-Jaschkowitz B, Turner CLS, Patalan M, Gizewska M, Binder G, Bich Ngoc CT, Chi Dung V, Mehta SG, Baynam G, Hamilton-Shield JP, Aljareh S, Lokulo-Sodipe O, Horton R, Siebert R, Elbracht M, Temple IK, Eggermann T and Mackay DJG. (2018). Maternal variants in NLRP and other maternal effect proteins are associated with multilocus imprinting disturbance in offspring. Journal of Medical Genetics 55, 497–504. 29574422 10.1136/jmedgenet-2017-105190 PMC6047157

[ref3] Begemann M, Spengler S, Kanber D, Haake A, Baudis M, Leisten I, Binder G, Markus S, Rupprecht T, Segerer H, Fricke-Otto S, Mühlenberg R, Siebert R, Buiting K and Eggermann T. (2011). Silver-Russell patients showing a broad range of IC1 and IC2 hypomethylation in different tissues. Clinical Genetics 80, 83–88. 20738330 10.1111/j.1399-0004.2010.01514.x

[ref4] Brioude F, Kalish JM, Mussa A, Foster AC, Bliek J, Ferrero GB, Boonen SE, Cole T, Baker R, Bertoletti M, Cocchi G, Coze C, De Pellegrin M, Hussain K, Ibrahim A, Kilby MD, Krajewska-Walasek M, Kratz CP, Ladusans EJ, Lapunzina P, Le Bouc Y, Maas SM, Macdonald F, Õunap K, Peruzzi L, Rossignol S, Russo S, Shipster C, Skórka A, Tatton-Brown K, Tenorio J, Tortora C, Grønskov K, Netchine I, Hennekam RC, Prawitt D, Tümer Z, Eggermann T, Mackay DJG, Riccio A and Maher ER. (2018). Expert consensus document: clinical and molecular diagnosis, screening and management of Beckwith-Wiedemann syndrome: an international consensus statement. Nature Reviews. Endocrinology 14, 229–249. 10.1038/nrendo.2017.166 PMC6022848 29377879

[ref5] Docherty LE, Rezwan FI, Poole RL, Turner CLS, Kivuva E, Maher ER, Smithson SF, Hamilton-Shield JP, Patalan M, Gizewska M, Peregud-Pogorzelski J, Beygo J, Buiting K, Horsthemke B, Soellner L, Begemann M, Eggermann T, Baple E, Mansour S, Temple IK and Mackay DJ. (2015). Mutations in NLRP5 are associated with reproductive wastage and multi-locus imprinting disorders in humans. Nature Communications 6, 8086. 10.1038/ncomms9086 PMC4568303 26323243

[ref6] Eggermann K, Bliek J, Brioude F, Algar E, Buiting K, Russo S, Tümer Z, Monk D, Moore G, Antoniadi T, Macdonald F, Netchine I, Lombardi P, Soellner L, Begemann M, Prawitt D, Maher ER, Mannens M, Riccio A, Weksberg R, Lapunzina P, Grønskov K, Mackay DJ and Eggermann T. (2016). EMQN best practice guidelines for the molecular genetic testing and reporting of chromosome 11p15 imprinting disorders: Silver-Russell and Beckwith-Wiedemann syndrome. European Journal of Human Genetics 24, 1377–1387. 27165005 10.1038/ejhg.2016.45 PMC5027690

[ref7] Murphy R, Mackay D and Mitchell EA. (2012). Beckwith Wiedemann imprinting defect found in leucocyte but not buccal DNA in a child born small for gestational age. BMC Medical Genetics 13, 99. 23116464 10.1186/1471-2350-13-99 PMC3514203

[ref8] Russo S, Calzari L, Mussa A, Mainini E, Cassina M, Di Candia S, Clementi M, Guzzetti S, Tabano S, Miozzo M, Sirchia S, Finelli P, Prontera P, Maitz S, Sorge G, Calcagno A, Maghnie M, Divizia MT, Melis D, Manfredini E, Ferrero GB, Pecile V and Larizza L. (2016). A multi-method approach to the molecular diagnosis of overt and borderline 11p15.5 defects underlying Silver-Russell and Beckwith-Wiedemann syndromes. Clinical Epigenetics 8, 23. 10.1186/s13148-016-0183-8 PMC4772365 26933465

[ref9] Sanchez-Delgado M, Riccio A, Eggermann T, Maher ER, Lapunzina P, Mackay DJG and Monk D. (2016). Causes and consequences of multi-locus imprinting disturbances in humans. Trends in Genetics 32, 444–455. 27235113 10.1016/j.tig.2016.05.001

[ref10] Soellner L, Begemann M, Degenhardt F, Geipel A, Eggermann T and Mangold E. (2017a). Maternal heterozygous NLRP7 variant results in recurrent reproductive failure and imprinting disturbances in the offspring. European Journal of Human Genetics 25, 924–929. 28561018 10.1038/ejhg.2017.94 PMC5567160

[ref11] Soellner L, Begemann M, Mackay DJ, Grønskov K, Tümer Z, Maher ER, Temple IK, Monk D, Riccio A, Linglart A, Netchine I and Eggermann T. (2017 b). Recent advances in imprinting disorders. Clinical Genetics 91, 3–13. 27363536 10.1111/cge.12827

[ref12] Tee L, Lim DH, Dias RP, Baudement MO, Slater AA, Kirby G, Hancocks T, Stewart H, Hardy C, Macdonald F and Maher ER. (2013). Epimutation profiling in Beckwith-Wiedemann syndrome: relationship with assisted reproductive technology. Clinical Epigenetics 5, 23. 10.1186/1868-7083-5-23 PMC3878854 24325814

[ref13] Turner CL, Mackay DJ, Callaway JL, Docherty LE, Poole RL, Bullman H, Lever M, Castle BM, Kivuva EC, Turnpenny PD, Mehta SG, Mansour S, Wakeling EL, Mathew V, Madden J, Davies JH and Temple IK. (2010). Methylation analysis of 79 patients with growth restriction reveals novel patterns of methylation change at imprinted loci. European Journal of Human Genetics 18, 648–655. 20104244 10.1038/ejhg.2009.246 PMC2987339

[ref14] Wakeling EL, Brioude F, Lokulo-Sodipe O, O'Connell SM, Salem J, Bliek J, Canton AP, Chrzanowska KH, Davies JH, Dias RP, Dubern B, Elbracht M, Giabicani E, Grimberg A, Grønskov K, Hokken-Koelega AC, Jorge AA, Kagami M, Linglart A, Maghnie M, Mohnike K, Monk D, Moore GE, Murray PG, Ogata T, Petit IO, Russo S, Said E, Toumba M, Tümer Z, Binder G, Eggermann T, Harbison MD, Temple IK, Mackay DJ and Netchine I. (2016). Diagnosis and management of Silver-Russell syndrome: first international consensus statement. Nature Reviews. Endocrinology 13, 105–124 10.1038/nrendo.2016.138 27585961

